# Anti-TGEV Miller Strain Infection Effect of *Lactobacillus plantarum* Supernatant Based on the JAK-STAT1 Signaling Pathway

**DOI:** 10.3389/fmicb.2019.02540

**Published:** 2019-11-06

**Authors:** Kai Wang, Ling Ran, Tao Yan, Zheng Niu, Zifei Kan, Yiling Zhang, Yang Yang, Luyi Xie, Shilei Huang, Qiuhan Yu, Di Wu, Zhenhui Song

**Affiliations:** ^1^Department of Microbiology and Immunology, College of Animal Science, Southwest University, Chongqing, China; ^2^Department of Preventive Veterinary Medicine, Medical College of Animals, Xinjiang Agricultural University, Ürümqi, China

**Keywords:** transmissible gastroenteritis virus, *Lactobacillus plantarum*, interferon-beta, STAT1, interferon-stimulating genes

## Abstract

Transmissible gastroenteritis (TGE), caused by transmissible gastroenteritis virus (TGEV), is one many gastrointestinal inflections in piglets, characterized by diarrhea, and high mortality. Probiotics are ubiquitous bacteria in animal intestines, which have many functions, such as promoting intestinal peristalsis and maintaining the intestinal balance. We found that the supernatant of the Lp-1 strain of *Lactobacillus plantarum*, isolated in our laboratory, and named Lp-1s had marked anti-TGEV effect on IPEC-J2 cells. Lp-1s could induce large amounts of interferon-β in IPEC-J2 cells in the early stage (6 h) of infection with TGEV, and increased the level of phosphorylated signal transducer and activator of transcription and its nuclear translocation in the late stage (24–48 h) of infection. This resulted in upregulated expression of interferon-stimulated genes, and increased the transcription and protein expression of antiviral proteins, resulting in an anti-TGEV effect.

## Introduction

Transmissible gastroenteritis virus (TGEV) is the pathogenic agent of porcine transmissible gastroenteritis (TGE), which causes vomiting, diarrhea, and high mortality in suckling piglets (Masters, 2006), resulting in heavy losses to the pig breeding industry (Zhao et al., 2014). In particular, viral diarrhea diseases are more serious because of limited treatment options. Probiotics comprise microorganisms that have beneficial activities to the host, and mainly comprise *Clostridium butyricum*, *Lactobacillus*, *Bifidobacteria*, Actinomycetes, and yeasts. They usually occupy the human gut and reproductive system, and can improve the balance of the host micro-ecology (Fuller, 1989; Maragkoudakis et al., 2010; da Silva Sabo et al., 2017; Stofilova et al., 2017). There is growing interest in the oral administration of appropriate probiotics to reduce the pressure in the intestines and produce an effective innate immune response (Pollmann et al., 2005; Maragkoudakis et al., 2010). In recent years, probiotic animal feed supplements have been developed as viable alternatives to antibiotics because of the ban on antibiotics in feed (Scharek et al., 2007). The addition of probiotic feed can prevent the infection of pathogens causing intestinal diseases, directly benefiting the animal host (Villena et al., 2014), or can indirectly enhance the host’s immune response by balancing the disordered microbiota (Lee et al., 2018). In addition, many basic and clinical studies have confirmed that probiotic strains have antiviral effects (Lee et al., 2011; Yuan et al., 2018). Studies have shown that *Lactobacillus plantarum* can stimulate the body’s innate and acquired immunity, and contributes to the production of inflammatory factors that inhibit the replication of the virus in the body. For example, *L. plantarum* strain YU (LpYU) not only has high interleukin (IL)-12-inducing activity mediated by Toll-like receptor (TLR) 2 in mouse peritoneal macrophages, but also communicates with natural killer cells (NK) in the spleen to stimulate the production of IgA to enhance the body’s anti-H1N1 virus activity (Kawashima et al., 2011). In addition, *L. plantarum* L-137 can stimulate the production of type I interferon (IFN-1) to effectively inhibit the proliferation of H1N1 (Maeda et al., 2009). TGEV is an important gastrointestinal diarrhea virus; therefore, research and exploration into the antiviral mechanism of probiotics could lead to the development of oral probiotics to prevent and treat TGEV infection.

The body reacts rapidly to viral invasion by synthesizing and secreting type I interferon IFN-1 (IFN-α/β), which plays a key role in the host antiviral response. The binding of IFN-1 to its receptor (interferon α/β receptor, IFNAR) leads to activation of the Janus family kinase (JAK) and subsequent signal transduction and transcriptional activator (STAT) signaling cascade, resulting in the activation and upregulation of interferon-stimulating genes (*ISGs*), ultimately activating IFN to exert its antiviral effects (Zhao et al., 2016; Chen et al., 2017).

A strain of *L. plantarum* was successfully isolated and named Lp-1 (Song Han et al., 2017). We wondered whether the IPEC-J2 cells treated by Lp-1 could induce an antiviral mechanism through the IFN-β/JAKs/STAT/*ISGs* pathway after TGEV infection. In the present study, we found that the supernatant of *L. plantarum* Lp-1 (Lp-1s) could significantly inhibit TGEV infection. By detecting the replication of TGEV N gene in porcine intestinal epithelial cells treated with Lp-1s at different time points, we confirmed that Lp-1s had a preventive effect against TGEV. Then, by detecting the levels of IFN, p-STAT and *ISGs*, we further confirmed that Lp-1s exerts its anti-TGEV role by upregulating the expression of IFN-β.

## Materials and Methods

### Cells, Viruses, and Reagents

Porcine kidney cells (ST) to amplify the virus and the experimental model pig jejunal cells (IPEC-J2 cells) were both cultured in Roswell Park Memorial Institute (RPMI) 1640 medium (Gibco, Grand Island, NY, United States) containing 10% fetal bovine serum (FBS, Gibco), under 37°C and 5% CO_2_. IPEC-J2 cells and ST cells were purchased from the Shanghai Sur Biotech Co., Ltd (Shanghai, China). *L. plantarum* LP-1 was isolated and stored in our laboratory. Its 16S ribosomal gene sequence has been submitted to GenBank (MH727586). Lp-1s was isolated from a culture of *L. plantarum* LP-1 after shaking for 14 h at 37°C. The TGEV Miller strain (Yang et al., 2018) was preserved in our laboratory. Viral fluid was collected from ST cells after replication for approximately 72 h when the cells showed obvious cytopathic effects (CPEs).

### Primer Design and Synthesis

The GenBank sequences of the TGEV N gene (GQ-374566.1) coding sequence (CDS) conserved region, the porcine MX1 CDS (MX dynamin like GTPase 1; AH015318.2), the MX2 CDS (MX dynamin like GTPase 2; AY897395.1), the ISG15 CDS (interferon-stimulated protein, 15 KDa; NM_214303.2), the OASL CDS (2′-5′-oligoadenylate synthetase like; NM_214303.2), the PKR CDS (double stranded RNA-dependent protein kinase; AB104654.1), the ZAP CDS (zeta-chain associated protein; GU_563332.1), and internal reference pig β-Actin gene (ACTB; XM_003124280.2) were obtained and pairs of specific primers were designed using Primer 5.0 software for quantitative real-time PCR [performed using SYBR Premix EX Taq II (Takara, Shiga, Japan)] as follows: TGEV-N (Forward: 5′-TTCAACCCC ATAACCCTCCAACAA-3′ and Reverse: 5′-GGCCCTTCAC CAT GCGATAGC-3′), MX1 (Forward: 5′-ATCTGTAAGCAGG AGACCATCAACTT G-3′ and Reverse: 5′-CTCGCCACGTCCA CTATCTTGTC-3′), MX2 (Forward: 5′-TTCACTCGCATCCGC ACTTCAG-3′ and Reverse: 5′-AGCTCCTCTGTCGCACTC TGG-3′), ISG15 (Forward: 5′-GGCAGCACAGTCCTGTT GATGG-3′ and Reverse: 5′-TGCGTCAGCCAGACCTCAT AGG-3′), OASL (Forward: 5′-CGTTGGTGGTGG AGACACA TACAG-3′ and Reverse: 5′-TCAGGCGACACCTTCCAGG ATC-3′), PKR (Forward: 5′-ACAGGACCTGCACATAACT TGAGG-3′ and Reverse: 5′-TGCTGTCGGCAGTGATGAAGA AC-3′), ZAP (Forward: 5′-GCTCAGTGCGAAC ACCTGGA TG-3′ and Reverse: 5′-TGACAGATGAAGGCGTGGAG AGG-3′), and ACTB (Forward: 5′-CTCTTCCAGC CCTCCT TCC-3′ and Reverse: 5′-GGTCCTTG CGGATGTCG-3′). The designed primers were synthesized by Shanghai Shenggong Biotechnology Service Co., Ltd. (Shanghai, China).

### Assessment of Cellular Toxicity of Lp-1s by the CPE Effect and the MTT Assay

After centrifugation of Lp-1 with an OD_600_ of 1.8 at 4000 rpm/min for 10 min, the obtained supernatant was filtered through a 0.22-μm filter, and then diluted with RPMI 1640 high sugar medium to six gradients at two times ratio, i.e., Lp-1s was diluted to obtain OD_600_ values of 0.9, 0.45, 0.225, 0.113, 0.056, and 0.028, respectively. IPEC-J2 cells were seeded at 1 × 10^5^/mL in 96-well plates and incubated overnight in 5% CO_2_ at 37°C. The medium was discarded when the cells reached 90% confluence in the 96-well plate, and then 100 μL of each gradient dilution of Lp-1s was added to each well. The medium in the control group was replaced with RPMI 1640. After incubating for 90 min, the supernatant was discarded, and the cells were washed twice with phosphate-buffered saline (PBS). Culture was continued and the cytopathic effect (CPE) was observed daily. The maximum non-toxic dose of Lp-1s to the cells was detected using the 3-(4,5-dimethylthiazol-2-yl)-2,5-diphenyltetrazolium bromide (MTT) reagent (BBI, Shanghai, China), when 80% of the cells in the negative control group were damaged. MTT assays for each dilution were repeated three times independently.

### Optimal Concentration of Lp-1s for Anti-TGEV Activity

Lp-1s was prepared as above and then diluted to an OD_600_ of 0.45, 0.225, 0.113, and 0.056 with RPMI 1640 high-sugar medium. IPEC-J2 cells were seeded at 1.8 × 10^6^/mL in 6-well plates and cultured in 5% CO_2_ at 37°C. At 90% confluence, the medium was discarded, the cells were washed three times with PBS, 1 mL of each gradient dilution of Lp-1s was added to each well, and the cells incubated at 37°C in 5% CO_2_ for 90 min. The medium in the control group was replaced with RPMI 1640. For the other wells, the supernatant was discarded, TGEV (multiplicity of infection (MOI) = 0.1) in RPMI 1640 medium was added to Lp-1s-treated IPEC-J2, and incubated at 37°C in 5% CO_2_ for 1.5 h. The supernatants were discarded and incubation continued in RPMI 1640 high glucose medium. After 48 h of culture, proteins were extracted for western blotting to detect the levels of the TGEV N protein after treatment with different concentrations of Lp-1s.

### Median Tissue Culture Infectious Dose (TCID50) Analysis

IPEC-J2 cells treated with Lp-1s for 1.5 h were exposed to TGEV (MOI = 0.1). The IPEC-J2 cells treated with Lp-1s were sampled at 12 h post infection (hpi), 24 hpi, and 48 hpi and then frozen and thawed three times to collect virus particles in the cells and supernatants. Gradient dilution of IPEC-J2 cells was performed from 10^–1^ and 10^–7^, respectively. TGEV titers of IPEC-J2 cells treated with Lp-1s for different times were detected using ST cells in 96-well plates. Each dilution gradient was assayed in 12 replicate wells. The TCID_50_ of the virus in the different groups was calculated by Reed and Muench methods.

### Detection of TGEV-N Gene Copy Number and Protein Expression

IPEC-J2 cells were inoculated into 6-well plates at 1.8 × 10^6^/mL. The IPEC-J2 cells treated with Lp-1s for 1.5 h were exposed to TGEV (MOI = 0.1) for RNA extraction and protein sampling. When the cells reached 90% confluence, RNA was extracted and reverse transcribed into cDNA and quantified at 500 ng/mL. Absolute fluorescence quantitative PCR was performed using fluorescence quantitative PCR. The reaction parameters were as follows: Pre-denaturation at 95°C for 3 min, followed by 40 cycles of denaturation at 94°C for 30 s, annealing at 60°C for 30 s, and prolongation at 72°C for 30 s. The reaction for each sample was repeated three times. The Bio-Rad CFX Manager random matrix method was used to analyze the linear relationship between cycle threshold (CT) value and the copy number to calculate the copy number of the TGEV-N gene. Protein samples were also extracted at the same time point and detected using western blotting. The primary antibody was a mouse monoclonal antibody against TGEV-N, and the secondary antibodies were horseradish peroxidase (HRP)-conjugated goat anti-mouse antibodies (Proteintech,Wuhan,China). The immunoreactive protein bands were visualized using a Vilber fusion FX5 chemiluminescent imager.

### Effect of Lp-1s on IFN-β Induction in IPEC Cells

IPEC-J2 cells were inoculated into 6-well plates at 1.8 × 10^6^/mL. When they reached 90% confluence, three experimental groups were established: An Lp-1s optimal concentration treatment group infected TGEV, a TGEV single infection group, and the uninfected control group. Cell samples at 6, 12, 24, and 48 hpi were centrifuged at 4000 rpm for 10 min, and the supernatant subjected to an enzyme linked immunosorbent assay (ELISA) to detect IFN-β.

### Western Blotting Analysis

Transmissible gastroenteritis virus (MOI = 0.1) was infected to IPEC-J2 cells that had been treated with Lp-1s for 1.5 h, and then cell samples at 12, 24, and 48 hpi were collected to produce protein lysates. In addition, TGEV (MOI = 0.1) was infected into IPEC-J2 cells cultured in RPMI 1640 for 1.5 h as the TGEV control group and the proteins were extracted at the same time points as the Lp-1s group. The protein concentration was determined using the bicinchoninic acid method and subjected to sodium dodecyl sulfate polyacrylamide gel electrophoresis. The separated proteins were transferred to a nitrocellulose membrane (Bio-Rad). The membranes were blocked using 5% skimmed milk and incubated with the following primary antibodies: Anti-STAT1 rabbit polyclonal antibodies (British Biorbyt Company), anti-phospho-(p)STAT1 (Tyr701) rabbit polyclonal antibodies (Biorbyt), anti-ZAP rabbit polyclonal antibodies (Abcam), anti-PKR rabbit polyclonal antibodies (Abcam), anti-(p)PKR (T446) rabbit polyclonal antibodies (Abcam), and anti-β-tubulin rabbit polyclonal antibodies (Proteintech). Secondary antibodies comprised goat anti-rabbit immunoglobulin (H + L). The immunoreactive protein bands were visualized using the Vilber fusion FX5 imaging system (VILBER), and the grayscale values of each band were analyzed by GraphPad Prism.

### Indirect Immunofluorescence Detection of Nuclear Displacement

IPEC-J2 cells were seeded at a density of 1.5 × 10^6^ in cell slides in 24-well culture dishes. These cells were set as three experimental groups comprising an Lp-1s optimal concentration treatment group, a TGEV alone infection group, and a blank (uninfected) control group, when they reached 90% confluence. The cells were sampled at 12, 24, and 48 hpi; washed three times with PBS; fixed with 4% paraformaldehyde at 37°C for 1.5 h; washed three times with PBS; permeated by 0.3% Triton-X-100 for 10 min; washed three times with PBS; and blocked by 1% bovine serum albumin for 30 min at room temperature. Primary antibodies comprising anti-p-STAT1 protein rabbit polyclonal antibodies and anti-TGEV-N mouse monoclonal antibodies were added and incubated overnight in 4°C in a wet box. The cells were then washed three times with PBS, proportionally added CY3-goat anti-rabbit fluorescence and fluorescein isothiocyanate (FITC)-labeled goat anti-mouse fluorescent secondary antibodies were then added, the cells were incubated for 1 h in a dark room at 37°C, incubated with 2-(4-amidinophenyl)-1H-indole-6-carboxamidine (DAPI) for 5 min, rinsed with PBS. Laser confocal microscopy was then used to observe p-STAT1, its nuclear translocation, and the TGEV N protein. The protein levels were analyzed using the ZEN software.

### Quantitative Real-Time Reverse Transcription Polymerase Chain Reaction (qRT-PCR)

IPEC-J2 cells were seeded at 1.8 × 10^6^/mL in 6-well plates, grown to 90% confluence, and then divided into three groups: Cells treated with Lp-1s for 1.5 h and then infected with TGEV (MOI = 0.1), TGEV infection alone, and the blank control (uninfected cells). The IPEC-J2 cells were sampled at 12, 24, and 48 hpi for qRT-PCR. Total RNA was extracted using the RNAiso plus reagent (Takara), and then single-stranded RNA was isolated using an RNA PCR (AMV) ver 3.0 kit (Takara). cDNA was then synthesized via reverse transcription. Quantitative real-time PCR was then performed using the SYBR premix EX Taq II (Takara) to detect the mRNA levels of ZAP, PKR, OASL, and ISG15 (fluorescent primers were synthesized by Shanghai Shenggong Bioengineering Technology Service Co., Ltd.).

### Small Interfering RNA (siRNA) Assays

Small interfering RNA (siRNA) targeting STAT1, 5′- GGAACAGAAATACACCTAT-3′ (produced by RIBO, China). Transfected with the STAT1-specific siRNA using LipofectamineTM 3000 (Invitrogen, United States), according to the manufacturer’s instructions, to the TGEV-infected groups treated with Lp-1s and TGEV infection alone groups, respectively, in IPEC-J2 cells. ConsiRNA transfected as control groups. The collected samples were analyzed by Western blotting using a rabbit pAb recognizing STAT1 as the primary antibody and HRP-conjugated goat anti-rabbit IgG as the secondary antibody.

### Statistical Analyses

All results were plotted and analyzed using GraphPad Prism 6 software (GraphPad Inc., La Jolla, CA, United States). The data were presented as the mean ± standard deviation (SD) of three independent experiments. Data were statistically compared using the *t* test. A *p* value < 0.05 (^∗^*p* < 0.05 and ^∗∗^*p* < 0.01) was considered statistically significant.

## Results

### MTT Cytotoxicity Test and Lp-1s Concentration Screening

**FIGURE 1:**
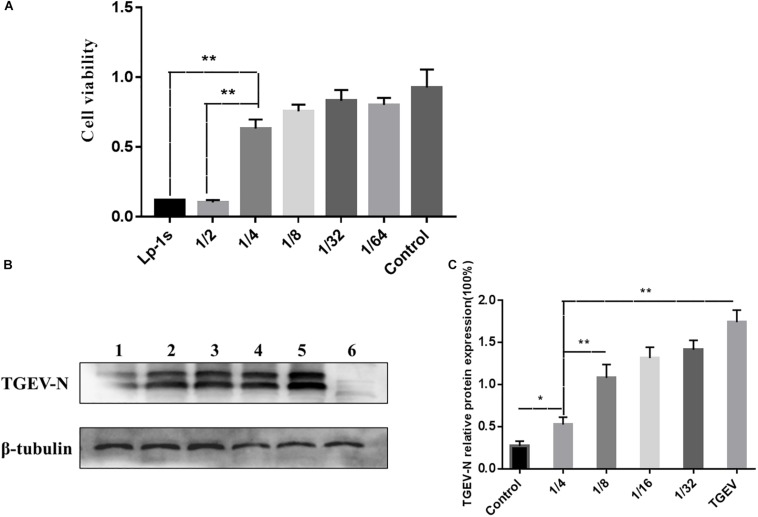
MTT cytotoxicity test and Lp-1s concentration screening. **(A)** Results of Lp-1s cytotoxicity as detected using the MTT method on 1/4, 1/8, 1/32, and 1/64 times dilutions of Lp-1s acting on IPEC-J2 cells. The cell adherence state remained basically unchanged. According to the experimental data, the Lp-1s undiluted group, the 1/2-fold dilution group and the 1/4-fold dilution group showed significantly difference in cytotoxicity (^∗∗^*P* < 0.01), and the 1/4-fold dilution group showed no significant difference compared with that of the control group (*P* > 0.05). **(B)** The expression of the TGEV N protein in IPEC-J2 cells treated with Lp-1s at different dilutions was detected by western blotting. Lane 1, infected TGEV group after Lp-1s 1/4 dilution pretreatment; Lane 2, infected TGEV group after Lp-1s 1/8 dilution pretreatment; Lane 3, infected TGEV group after Lp-1s 1/16 dilution pretreatment; Lane 4, infected TGEV group after Lp-1s 1/32 dilution pretreatment; Lane 5, TGEV infection group; and Lane 6, uninfected control cells (normal group). **(C)** Grayscale analysis of the relative expression of TGEV N in IPEC-J2 cells infected with Lp-1s at different dilutions showing that TGEV N protein levels were decreased in the 1/4-fold dilution of Lp-1s treatment group after 48 h, compared with the 1/8-fold dilution and TGEV infection group, there was a significant difference (^∗∗^*P* < 0.01), which was significantly different from the control group (^∗^*P* < 0.05).

The MTT assay (Figure 1) showed that the higher dilution ratio of Lp-1s, the higher the cell viability. The maximum non-toxic dose toward the cells was greater than 50% OD_490_; therefore, the maximum non-toxic dose of Lp-1s to IPEC-J2 cells is OD_600_ 0.45 (Figure 1A). The TGEV inhibition rate of Lp-1s decreased with increasing dilution factor and was thus concentration dependent. The results showed that amount of TGEV Miller strain (MOI = 0.1) was reduced by 1/4-fold by Lp-1s, i.e., the OD_600_ was 0.45, and the treated IPEC-J2 cells received the highest non-toxic dose of Lp-1s (Figure 1B).

### Results of TCID_50_ Experiment

**FIGURE 2:**
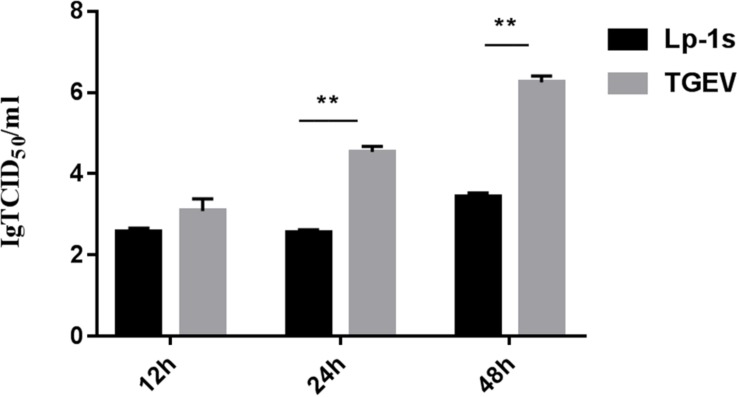
TCID_50_ analysis. The results of statistical analysis showed that the 24 h and 48 h Lp-1s supernatant treatment groups had significantly different TCID_50_ values compared with that of the simultaneous TGEV challenge group (^∗∗^*P* < 0.01) demonstrating that IPEC-J2 had a reduced viral titer of the TGEV Miller strain after treatment with Lp-1s.

The experimental data (Figure 2) showed that the TGEV titers at 12, 24, and 48 hpi after TGEV infection of IPEC-J2 cells treated with Lp-1s were 10^2.5^ TCID_50_/mL, 10^2.55^ TCID_50_/mL, and 103.3 TCID_50_/mL, respectively. Compared with the TGEV control group, the amount of viral of lesions decreased by 1.1, 1.9, and 1.8-fold at 12, 24, and 48 hpi, respectively, in the Lp-1s group and no lesions appeared in the blank control group. Therefore, Lp-1s has a significant anti-TGEV effect (^∗∗^*p* < 0.01) and is optimal for antiviral activity at 24 hpi.

### TGEV-N Gene Copy Number and Protein Expression Levels in Response to Lp-1s

**FIGURE 3:**
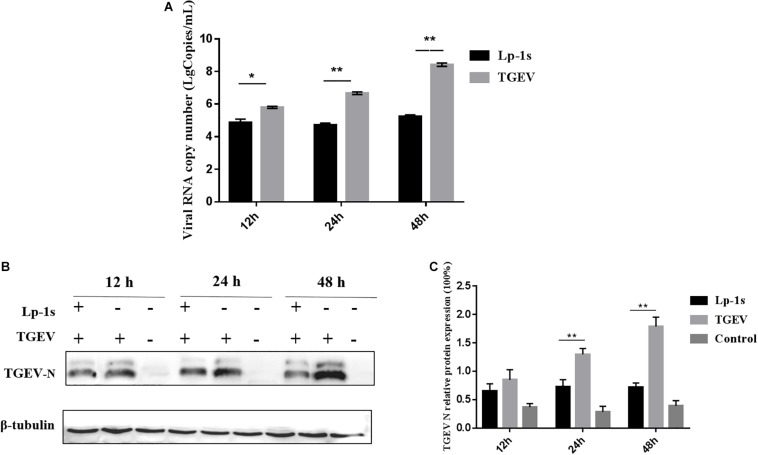
Time course of TGEV N gene and protein expression. **(A)** TGEV N gene copy number in different groups of cells infected with TGEV for 12, 24, and 48 h. The TGEV N gene copy number was significantly different between the TGEV group and the TGEV group after treatment with Lp-1s for 12 h (^∗^*P* < 0.05). The TGEV group and the TGEV group with Lp-1s treatment infected for 24 and 48 h showed a significant difference (^∗∗^*P* < 0.01). **(B)** Western blotting detection of TGEV N protein levels at different time points after infection of TGEV in Lp-1s-treated cells. **(C)** Grayscale analysis of TGEV N protein levels at different time points after infection of TGEV by Lp-1s treated cells. There was no significant difference between the two groups (*P* > 0.05) at 12 h; however, the difference was extremely significant at 24 and 48 h (^∗∗^*P* < 0.01). Lp-1s, Lp-1s pretreatment of IPEC-J2 cells infected with TGEV; TGEV, cells directly infected with TGEV.

We found that the TGEV N gene copy number in IPEC-J2 cells treated with Lp-1s was lower than that in cells infected with TGEV only (^∗^*p* < 0.05, ^∗∗^*p* < 0.01). In addition, the viral N gene copy number in the TGEV infection group was positively correlated with the infection duration (Figure 3A). Western blotting showed that the level of the TGEV N protein in the Lp-1s group was lower than that in the TGEV group at all three time points. No expression of TGEV N protein was observed in the blank control group (Figures 3B,C). These results demonstrated that Lp-1s could inhibit the transcription and protein expression of TGEV N.

### Lp-1s Induces IFN-β

**FIGURE 4:**
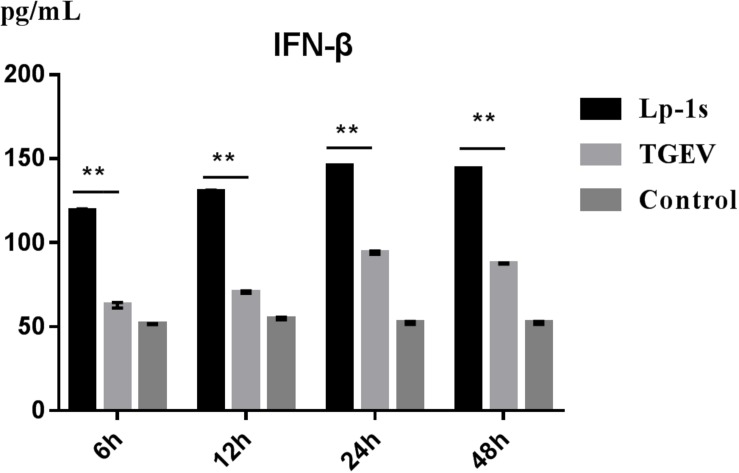
Changes in IFN-β levels induced by cells in different groups. Lp-1s, Lp-1s pretreatment of IPEC-J2 cells infected with TGEV; TGEV, cells directly infected with TGEV; Con, uninfected cells (normal group). There were significant differences in IFN-β levels between the TGEV group and the Lp-1s group at different time points (^∗∗^*P* < 0.01).

The results in Figure 4 indicate that IFN-β levels gradually increased in IPEC-J2 cells during TGEV infection. However, compared with the TGEV-infected group, the IFN-β level of the TGEV (MOI = 0.1)-infected group treated with Lp-1s increased significantly with infection time (^∗∗^*p* < 0.01). Therefore, Lp-1s can significantly increase the level of intracellular IFN-β during TGEV infection.

### Lp-1s Stimulates the Phosphorylation of STAT1

**FIGURE 5:**
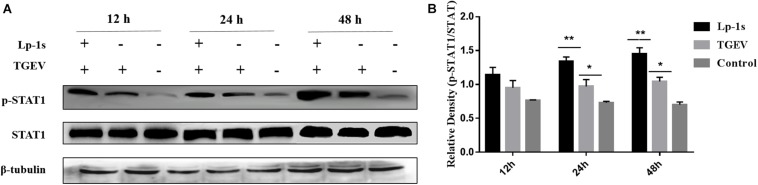
Western blotting detection of p-STAT1 and STAT1 levels in different groups. Lp-1s, Lp-1s pretreatment of IPEC-J2 cells infected with TGEV; TGEV, cells directly infected with TGEV; Con, uninfected cells (normal group). **(A)** STAT1 total protein levels and phosphorylated STAT1 levels at 12–48 h. **(B)** Grayscale analysis of the levels of phosphorylated STAT1 in the different groups. Statistical analysis showed that the level of phosphorylated STAT1 was higher in the Lp-1s group than in the TGEV group after 12 h (*P* > 0.05); after 24 h of Lp-1s treatment, the level of phosphorylated STAT1 in the Lp-1s group was significantly higher than that in the TGEV group (^∗∗^*P* < 0.01); after 48 h of Lp-1s treatment, the level of phosphorylated STAT1 in the Lp-1s group was significantly higher than that in the TGEV group (^∗^*P* < 0.05). The results showed that treatment of TGEV-infected IPEC-J2 cells with Lp-1s (24–48 h), induced increased of levels of phosphorylated intracellular STAT1.

The results in Figure 5 show that there was almost no change in the total expression of STAT1 in IPEC-J2 cells under different treatments and at different time points; however, the amount of phosphorylated STAT1 changed significantly. The amount of phosphorylated STAT1 in the Lp-1s group was higher than that in the TGEV group at the different time points (^∗^*p* < 0.05 and ^∗∗^*p* < 0.01), while only a small amount of phosphorylated STAT1 was detected in the blank control group at the different time points. Therefore, although TGEV infection could significantly increase the amount of phosphorylated STAT1, Lp-1s could further significantly increase the amount of phosphorylated STAT1 in cells after TGEV infection.

### Lp-1s Activates Nuclear Translocation of p-STAT1

**FIGURE 6:**
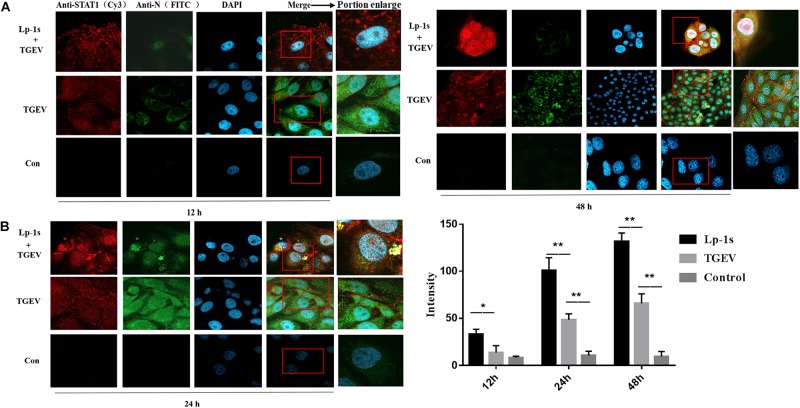
Identification of nuclear translocation in porcine p-STAT by IFA (magnification: 63×). Lp-1s, Lp-1s pretreatment of IPEC-J2 cells infected with TGEV; TGEV, cells directly infected with TGEV; Con, uninfected cells (normal group). **(A)** Anti-pSTAT1 (Cy3), goat anti-rabbit IgG Cy3 labeled p-STAT1 protein; Anti-TGEV N (FITC), goat anti-mouse IgG FITC labeled TGEV N protein; DAPI, DAPI nuclear staining; Merge, fused image of the three fluorescent images. **(B)** The nucleus was identified using Zen blue software and the intensity of red fluorescence emitted by Cy3-labeled p-STAT1 was analyzed. The statistical results showed that the fluorescence intensity of Cy3 in IPEC-J2 cells treated with Lp-1s was significantly higher than that in the TGEV group after 12 h (^∗^*P* < 0.05). In the late stage of TGEV infection (24–48 h), the fluorescence signal intensity of p-STAT1 in the nucleus of this group was significantly higher than that in TGEV group (^∗∗^*P* < 0.01).

The results in Figure 6 show that the amount of p-STAT1 (red fluorescence) in the nuclei of TGEV infected cells treated with Lp-1s at different time points was significantly higher than that in the nuclei of cells in TGEV infected group. At the same time, the amount of p-STAT1 correlated positively with the duration of infection after Lp-1s-treatment of IPEC-J2 cells infected with TGEV. However, the amount of red fluorescence emitted by p-STAT1 labeled with Cy3 correlated negatively with the amount of green fluorescence emitted by FITC-labeled TGEV N protein. We found that when TGEV was directly infected into IPEC-J2 cells, the red fluorescence of p-STAT1 was mainly gathered around the cell wall and only a small amount appeared in the nucleus; the green fluorescence and red fluorescence of the blank control group were not obvious. Meanwhile, the signal intensity of p-STAT1 in the nuclei of the Lp-1s treatment group correlated positively with time (Figure 6). Therefore, Lp-1s could significantly increase the level of p-STAT1 and promoted its translocation into the nucleus, while simultaneously inhibiting the expression of TGEV N.

### Transcriptional Expression of *ISGs*

**FIGURE 7:**
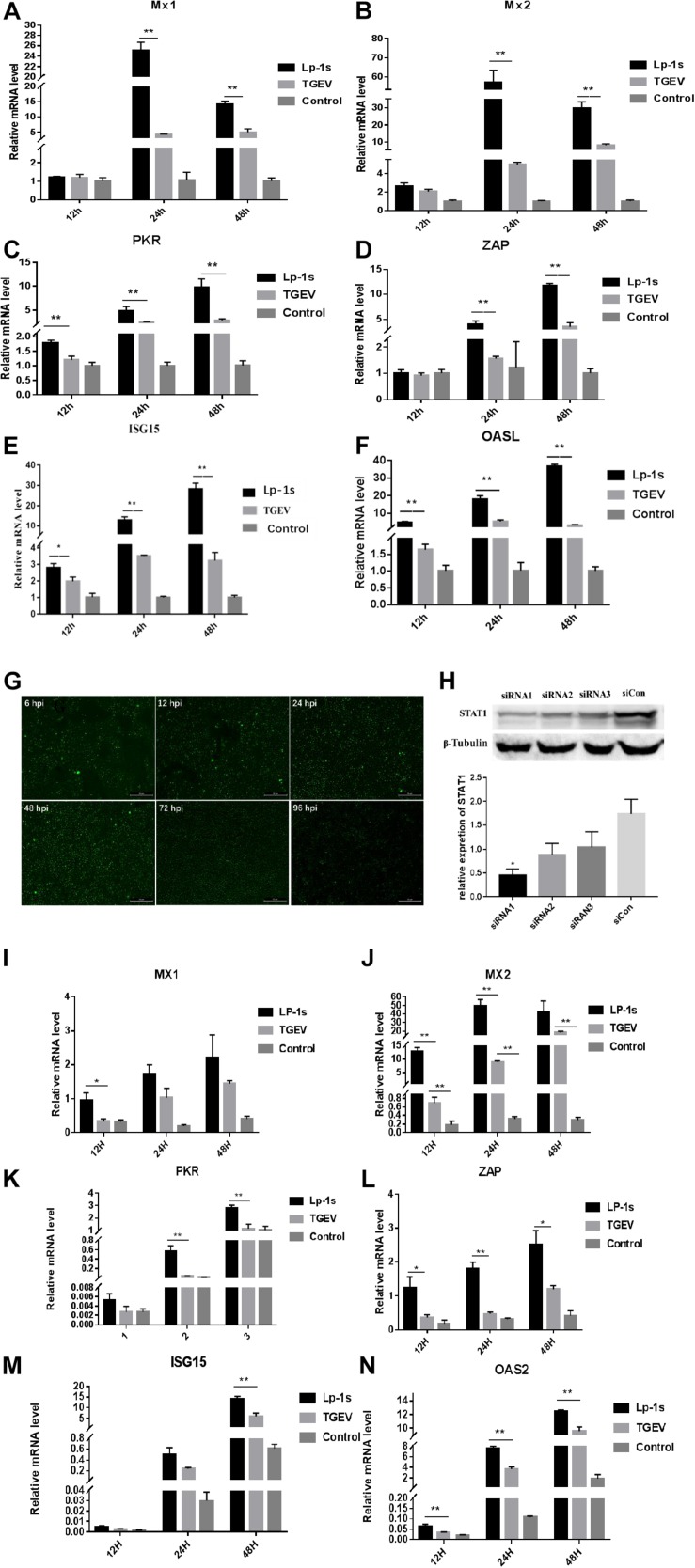
Relative mRNA expression levels of *ISGs* in different experimental groups. Lp-1s, Lp-1s pretreatment of IPEC-J2 cells infected with TGEV; TGEV, cells directly infected with TGEV; Con, uninfected cells (normal group). **(A–F)** Relative mRNA expression levels of *MX1*, *MX2*, *PKR*, *ZAP*, *ISG15*, and *OASL*, respectively. The expression levels of *ZAP*, *MX2*, and *MX1* in Lp-1s group after 12 h were not significantly different from those in the TGEV group (*P* > 0.05). The expression level of *ISG15* was significantly higher in the Lp-1s group than in the TGEV group (^∗^*P* < 0.05). The expression levels of *PKR* and *OASL* were significantly higher in the Lp-1s group than in the TGEV group (^∗∗^*P* < 0.01). The expression levels of *ZAP*, *MX2*, *MX1*, *PKR*, *OASL*, and *ISG15* in the Lp-1s group after 24 and 48 h were significantly higher than those in the TGEV group (^∗∗^*P* < 0.01). **(G,H)** Screening of siRNA targeting STAT1 optimal treatment time and gene silencing efficiency fragment. As shown in the figure, the optimal time for siRNA targeting STAT1 is 48 h, and the best gene silencing siRNA fragment is siRNA1 (^∗^*P* < 0.05). **(I–N)** Relative mRNA expression levels of *MX1*, *MX2*, *PKR*, *ZAP*, *ISG15*, and *OASL*, respectively, after targeting gene silencing STAT1. The expression levels of *PKR* and *OASL* in Lp-1s group after 12 h were not significantly different from those in the TGEV group (*P* > 0.05). The expression level of *MX2*, *PKR*, *ZAP*, *ISG15*, and *OASL* was significantly higher in the Lp-1s group after 24 h than in the TGEV group (^∗^*P* < 0.05 and ^∗∗^*P* < 0.01). The expression levels of *ZAP*, *ISG15* and *OASL* were significantly higher in the Lp-1s group after 48 h than in the TGEV group (^∗^*P* < 0.05 and ^∗∗^*P* < 0.01).

We found that the mRNA expression levels of *ZAP*, *MX2*, *MX1*, *PKR*, *OASL*, and *ISG15* were significantly higher in the Lp-1s treated group than in the TGEV infected group at various points after infection. The expression levels of *ZAP*, *PKR*, *OASL*, and *ISG15* in each experimental group increased with time. The expression levels of *MX1* and *MX2* peaked at 24 h and then decreased at 48 h (Figures 7A–F). The results showed that the best time to STAT1-siRNA targeting gene silencing STAT1 was 48 h (Figure 7G), and the best interference fragment was STAT1-siRNA1 (Figure 7H). After gene silencing STAT1, we found that the expression of ISGs decreased compared to the groups which no knock down STAT1, and the expression ISGs of Lp-1s-treated TGEV infected group was higher than that of TGEV infected alone group (Figures 7I–N). The results showed that TGEV infection of IPEC-J2 cells treated with Lp-1s could stimulate the expression of *ISGs* in cells to inhibit viral replication.

### Proteins Levels of ZAP, PKR, and p-PKR

**FIGURE 8:**
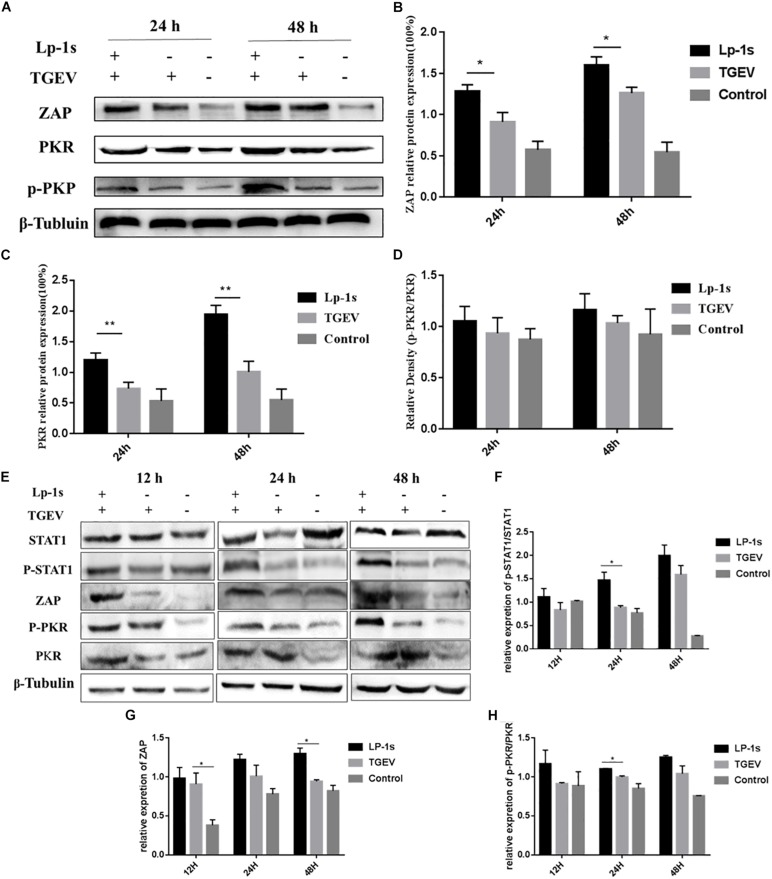
Protein levels of ZAP, PKR and p-PKR in different experimental groups. Lp-1s, Lp-1s pretreatment of IPEC-J2 cells infected with TGEV; TGEV, cells directly infected with TGEV; Con, Uninfected cells (normal group). **(A)** The expression of ZAP, PKR, p-PKR, and β-Tubulin protein. **(B)** Grayscale analysis of the ZAP/β-Tubulin results. The ZAP level of Lp-1s treatment group was significantly higher than TGEV infected alone at 24 and 48 h (^∗^*P* < 0.05). **(C)** Grayscale analysis of the PKR/β-Tubulin ratio. The ratio of PKR/β-Tubulin in the Lp-1s group was significantly higher after 48 h compared with that in the TGEV group (^∗∗^*P* < 0.01). **(D)** Grayscale analysis of the p-PKR/PKR ratio. There was no significant difference in the p-PKR/PKR ratio at the different time points (*P* > 0.05). **(E–H)** The expression of STAT1, p-STAT1, ZAP, PKR, and p-PKR proteins after silencing STAT1. **(E)** The expression of STAT1, p-STAT1, ZAP, PKR, p-PKR, and β-Tubulin protein. **(F)** Relative expression of p-STAT1/STAT1 in Lp-1s treatment group was significantly higher than TGEV infected alone at 24 h (^∗^*P* < 0.05). **(G)** Relative expression of ZAP in Lp-1s treatment group was significantly higher than TGEV infected alone at 48 h (^∗^*P* < 0.05). **(H)** Relative expression of p-PKR/PKR in Lp-1s treatment group was significantly higher than TGEV infected alone at 24 h (^∗^*P* < 0.05).

As expected, the protein levels of ZAP, PKR, p-PKR (Figures 8A–D) at 24, and 48 hpi in the Lp-1s group were significantly higher than those in negative control group and TGEV group (ZAP, ^∗^*P* < 0.05 and PKR, ^∗∗^*P* < 0.01). There was no significant change in the relative expression of p-PKR/PKR, however, the expression level of p-PKR protein varied with the amount of PKR protein. The level of STAT-1 in Figure 8E where the expression of this gene was knocked down using siRNA is significantly lower than the level of unknocked down STAT1 in Figure 5A. After gene silencing STAT1, the protein levels of STAT1, p-STAT1 ZAP, PKR, p-PKR (Figures 8E–H) at 12, 24, and 48 hpi in the Lp-1s group were significantly higher than TGEV group (p-STAT1/STAT1, ^∗^*P* < 0.05; ZAP, ^∗^*P* < 0.05; and p-PKR/PKR, ^∗^*P* < 0.05). The results showed that Lp-1s could increase the expression of *ISGs* and inhibit the replication of TGEV in IPEC-J2 cells, which was basically consistent with the expression trend of the *PKR* and *ZAP* genes.

## Discussion

Previous studies have found that many lactic acid bacteria can inhibit the infection of diarrhea-causing viruses (such as RV and PEDV) in the host through a variety of methods (Hou et al., 2007; Kawakami et al., 2010). Some lactic acid bacteria can be recognized by Toll like receptor (TLR)-2 or TLR-9 to enhance their response to stimulation by the interferon inducer poly (I: C) and induce a large amount of IFN-I (Maeda et al., 2009; Kanmani and Kim, 2018; Lee et al., 2019). At the same time, they can also upregulate the transcription of *IL6* and *TNFA* (Reyes-Diaz et al., 2018). In addition, some lactic acid bacteria can enhance the expression of surface molecules and cytokines in intestinal antigen presenting cells (APC), and enhance the molecular expression of MHC-II and IL-1β (Villena et al., 2014). In addition, other lactic acid bacteria can also stimulate the response level of TLR-3 to poly (I: C) (Hosoya et al., 2011). They can also regulate the role of TLR-2, TLR-4, and TLR negative regulators in the immune response, further enhancing the production of IFN-I induced by cells, and upregulate the transcription level of related antiviral factors (such as *MXA* and *OASL*) (Castillo et al., 2011). Other studies have shown that probiotics can inhibit TGEV infection by adsorbing virus particles and stimulating cells to produce innate immunity (Chai et al., 2013).

Recent studies have shown that the main reasons why the body’s interferon-beta (IFN-β) cannot fully exert its antiviral effect after TGEV infection are as follows: First, the cells do not respond in time to the immune response because of the level of viral replication and the virus titer of TGEV in the early stage of infection, resulting in lower levels of IFNs, which is the main cause of the short burst of TGEV latency (Zhu et al., 2017). Second, IFN-β does not play a direct antiviral role. Its antiviral function is produced by activating the IFN-mediated JAK-STAT signaling pathway to stimulate downstream interferon-stimulating genes (*ISGs*) (Saha and Pahan, 2006; Li, 2008; Proia et al., 2011). And, activation of the JAK-STAT1 signaling pathway requires Phosphorylated STAT1 (Tyr 701) enters the nucleus to activate interferon-stimulating factors *ISGs* (including MX1, MX2, PKR, OAS, ISG15, and ZAP) (Hovanessian, 1991; Zhu et al., 1997; Shi et al., 2010; Goujon et al., 2013; Amici et al., 2015; Li et al., 2015; Nigg and Pavlovic, 2015). ZAP can bind viral RNA directly and prevent the accumulation of viral RNA in the cytoplasm. It can also recruit RNA exosomes to degrade target viral RNA (Li et al., 2015). PKR-mediated inhibition of viral replication is activated by the formation of dsRNA during the replication of single-stranded RNA after viruses invade cells. The main reason is that the amino terminus of PKR can recognize the dsRNA domain and the carboxyl terminus has the kinase domain. When the viral double-stranded RNA is recognized, the inactive PKR protein located in the cytoplasm is phosphorylated. On the other hand, it can also regulate the cell immune response and autophagy caused by virus invasion to inhibit the virus. At the same time, PKR can activate the nuclear factor kappa B (NF-kB) signaling pathway via phosphorylation and further induce IFN production in cells (Sudhakar et al., 2000; Amici et al., 2015).

In the present study, we found that IPEC-J2 cells still produced IFN-β after infection with TGEV, which increased with time, reaching a peak at 24 h and no longer increased at 48 h. At the corresponding time points, the level of infection, viral titer, and replication of TGEV on IPEC-J2 cells showed an increasing trend. After Lp-1s treatment, IPEC-J2 cells produced a large amount of IFN-β at the early stage of TGEV infection (6 h), which was significantly higher than that of cells infected with TGEV only. The induced level of IFN-β in Lp-1s-treated IPEC-J2 cells was significantly different from that in the TGEV infected group at the same time point. The induction level of IFN-β in the Lp-1s treated group was significantly higher than that in TGEV infected group at the different time points. The level of IFN-β induction correlated positively with time, peaking at 24 h before decreasing toward 48 h. The early boost in IFN-β production in IPEC-J2 cells treated with Lp-1s might be one of the reasons for its inhibition of TGEV.

In addition, although TGEV delayed the expression of IFN-β in the early stage of infection, it promoted the expression of IFN-β at the peak of viral replication. The expression of IFN-β was parallel to the increase of viral RNA replication level at 24–48h, which demonstrated that TGEV replication remained high in the late stage of infection when IFN-β was produced in large quantities. This might be caused by the inhibitory effect of TGEV on IFN-β-mediated signaling pathways, resulting in the inability of IFN-β to regulate the transcription and expression of downstream target cytokines and exert an antiviral role.

Although there was no significant difference in the levels of p-STAT1 between the Lp-1s group and the TGEV group at 12 hpi, the level of p-STAT1 in the Lp-1s group was significantly higher than that in the TGEV group at the later stages (24 and 48 h). The level of p-STAT1 in the Lp-1s group correlated positively with time from 12–48 h. The results showed that IPEC-J2 cells treated with Lp-1s could effectively increase STAT1 phosphorylation in the late stage of virus infection. At the same time, the level of p-STAT1 changed slightly from 12–48 h after infection with TGEV. The results were quite different from those reported in previous studies on the infection of ST cells by TGEV. In addition, the level of p-STAT1 in IPEC-J2 cells infected with TGEV from 24–48 h was significantly lower than that in ST cells infected with TGEV from 24–48 h, which might reflect the difference between the cell lines and viruses used.

Further experiments showed that the level p-STAT1 nuclear translocation in the Lp-1s group was significantly higher than that in the TGEV group at the same time points, and p-STAT1 nuclear accumulation correlated positively with time. In TGEV-infected cells, p-STAT1 at the late stage of infection (24–48 h) accumulated in large amounts near the cell membrane and only a few nuclear translocations occurred. We speculated that the reason might be that the intracellular STAT1 protein is activated by IFN-β after IPEC-J2 cells are directly infected with TGEV to form a homologous or heterodimer, and the virus interacts with its receptor IRF9. The interaction resulted in the inability of activated STAT1 to undergo nuclear translocation through receptor-induced endocytosis, and could only dissociate around IFNAR, which reduced the JAK-STAT signaling pathway cascade response and antagonized the antiviral effect of IFN-β. The fluorescence value of activated STAT1 was significantly higher than that of blank control group. This indicated that TGEV could not completely escape the immune mechanism of IFN-β production in IPEC-J2 cells. At the same time, the fluorescence intensity of p-STAT in the nucleus of the cells in the Lp-1s treatment group correlated positively with time, and the intensity of FITC-labeled TGEV N protein was significantly lower than that in the TGEV treatment group at the same time point. These results showed that IPEC-J2 cells treated with Lp-1s could indeed activate the JAK-STAT1 signaling pathway when infected with TGEV, and the intensity of JAK-STAT1 signaling pathway was significantly higher in the Lp-1s-treated cells than in the cells directly infected with TGEV. As the signaling pathway cascade response increased, the replication level of TGEV in IPEC-J2 cells was further inhibited.

Finally, the transcriptional levels of *ISGs* in the Lp-1s treatment group were different at different time points. *MX1* and *MX2* reached their peak at 24 h, while the transcriptional levels of the two *ISGs* decreased at 48 h after TGEV infection. The transcription levels of *PKR*, *ZAP*, OASL and *ISG15* were positively correlated in groups. We speculated that the decrease in *MX1* and *MX2* mRNA transcription levels within 48 h after Lp-1s treatment might be related to the cycle of infected cells. The expression of *ISGs* of gene silenced STAT1 decreased as a whole compared to the groups which no knock down STAT1, indicating that STAT1 could affect downstream *ISGs*. While the *ISGs* of TGEV infected group treated with Lp-1s showed an upward trend, which further confirmed that Lp-1s could activate downstream *ISGs*. To further explore the difference in the intracellular response of Lp-1s treated IPEC-J2 cells to TGEV infection, we detected the changes in ZAP and PKR protein levels. The level of the ZAP protein in the Lp-1s and TGEV groups was consistent with its transcription level at the time point. Although the level of the ZAP protein in Lp-1s treated cells was significantly higher than that in the TGEV infection group, the difference in the transcription level of *ZAP* was significantly lower than that in the TGEV infection group. These results suggested that after Lp-1s treatment, the IFN-β and JAK-STAT1 signaling pathways induced in the cells are enhanced, and the expression of the ZAP protein is still limited, suggesting that other factors have an impact on the transcriptional regulation of ZAP. The protein level of PKR was consistent with its mRNA transcription level. There was no significant difference in the p-PKR/PKR values between the two groups at 24 and 48 h; however, the p-PKR/β-tubulin protein values were in line with our expectations. The results showed that the p-PKR level in the Lp-1s group was significantly higher than that in the TGEV group, and correlated positively with time. Therefore, we believe that the increase in p-PKR is mainly determined by the expression of the PKR protein.

**FIGURE 9:**
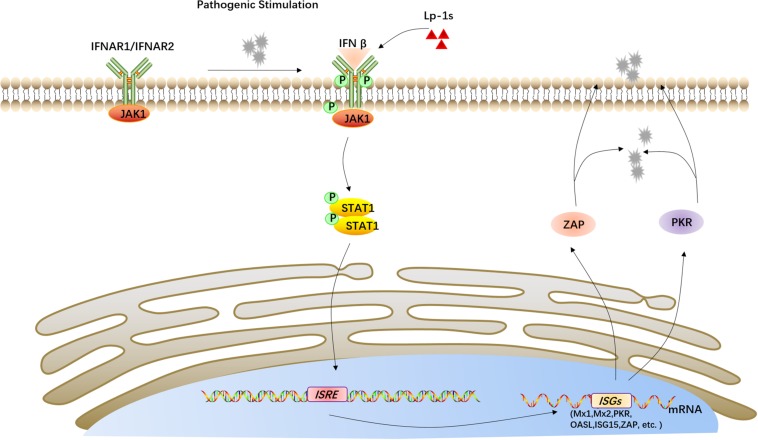
Proposed mechanism of the anti-TGEV effect of Lp-1s. Lp-1s increases IFN-β expression, and the binding of IFN-β to its receptor IFNAR leads to activation of the Janus family kinase (JAK) and subsequent activation of signal transduction and transcriptional activator 1 (STAT1) signaling cascades. These signaling pathways upregulate downstream interferon-stimulated genes (*ISGs*), including *MX1*, *MX2*, *PKR*, *OAS*, *ISG15*, and *ZAP*), which produce the corresponding antiviral proteins, e.g., ZAP and PKR, ultimately activating IFN-β to exert an antiviral effect.

Based on the above results, Lp-1s plays an antiviral role by stimulating the IFN-β-mediated JAK1/STAT pathway, resulting in upregulation interferon-stimulating genes, which induce the synthesis of antiviral proteins such as ZAP and PKR (Figure 9).

## Data Availability Statement

The datasets generated for this study are available on request to the corresponding author.

## Author Contributions

KW and ZS conceived and designed the experiments. KW, LR, and TY performed the experiments. ZN, ZK, YZ, YY, LX, SH, QY, and DW analyzed the data. LR and ZS wrote the manuscript.

## Conflict of Interest

The authors declare that the research was conducted in the absence of any commercial or financial relationships that could be construed as a potential conflict of interest.
